# Examining continuance intention of online learning during COVID-19 pandemic: Incorporating the theory of planned behavior into the expectation–confirmation model

**DOI:** 10.3389/fpsyg.2022.1046407

**Published:** 2022-11-17

**Authors:** Li Li, Qing Wang, Jinhui Li

**Affiliations:** ^1^School of Journalism, Yunnan University, Kunming, China; ^2^School of Journalism and Communication, Jinan University, Guangzhou, China; ^3^National Media Experimental Teaching Demonstration Center, Jinan University, Guangzhou, China

**Keywords:** online learning, coronavirus disease, expectation-confirmation model, the theory of planned behavior, China

## Abstract

COVID-19 pandemic has tremendously affected many industries in the world, including higher education. To cope with changes in the pandemic, online learning has become a prevalent means for university students to receive education. Based on the expectation-confirmation model (ECM) and the theory of planned behavior (TPB), this study aims to develop an integrated model, consisting of confirmation, perceived usefulness, satisfaction, perceived enjoyment, subjective norm, perceived behavioral control, and continuance intention instruments, to predict Chinese university students’ continuance intention toward online learning. Using convenience sampling, we enrolled full-time university students who have used online-learning platforms in November 2020. Data collected from 493 Chinese university students were analyzed by confirmatory factor analysis (CFA) and structural equation model (SEM) to test the model and hypotheses. All measurements of constructs used in this study are adapted by previous studies. The results show that perceived satisfaction, subjective norms, and perceived behavioral control were significantly associated with the continuance intention of online learning. Furthermore, the two components of the expectation–confirmation model (ECM), perceived usefulness and perceived enjoyment, have a significant impact on the satisfaction of online learning. Notably, although confirmation of expectations had no direct impact on satisfaction, it was positively associated with perceived usefulness and perceived enjoyment. Implications and limitations were discussed.

## Introduction

During the pandemic of Coronavirus Disease 2019 (COVID-19), many countries closed down their traditional face-to-face education systems to reduce the possibilities of virus transmission. In response to the changes in the pandemic, using online teaching models to replace physical teaching became a universal trend, especially in higher education. Since COVID-19 was first reported, many researchers have focused on its effects on online learning, including opportunities and challenges (Flores and Swennen, 2020; Yan et al., 2021; Chiu, 2022). An empirical study from Canada (Lemay et al., 2021) showed that the social distancing and isolation turned to be the barriers to effectively adopt online learning during the pandemic. These results are consistent with a Korean study, which indicated that the online-learning method may create loneliness, anxiety, and stress among exchange students (Stewart and Lowenthal, 2022). Moreover, due to the lack of courses learning value and fun, the negative attitude toward online learning has been discovered among American undergraduates (Garris and Fleck, 2022). Other factors, such as the familiarity of technology-based education and the accessibility of online learning, could also influence the continuance intention of online learning (Adnan and Anwar, 2020; Xhelili et al., 2021). Some scholars have pointed out that the online course quality and system quality (Li et al., 2021) may influence students perceived usefulness of this method, which, in turn, affect the continuous and actual use of online learning in the post-COVID-19 era (Mustafa and Garcia, 2021).

In China, guided by the idea of “Classes suspended but learning continues,” the Chinese Ministry of Education has promoted a number of online courses and online-learning platforms to maintain learning activities in the past 3 years. Students are now able to adopt online-learning platforms to receive education at any time and from anywhere without being limited by time and space (Vlachopoulos, 2020). As of June 2021, there are 325 million online education users in China, accounting for 32.1% of the total population (China Internet Network Information Center, 2021). Although online learning has many advantages, some recent studies have found that its rate of completion and effectiveness has not significantly increased during the pandemic (Liu et al., 2020; Yang et al., 2021). Because online learning is becoming growingly important in advanced education, it is crucial to find out what factors influence university students’ continuous intention to adopt online-learning method.

Recently, some research have examined Chinese students’ intention to continue learning online (Dai et al., 2020). However, it remains unclear what factors influence students’ continuation intentions toward online learning. This study aims to bridge this gap by investigating the factors affecting Chinese college students’ continued intention of using online learning from a theoretical perspective. Considering that the Expectation-Confirmation Model (ECM) has become the most applied theory to explain individuals’ usage behaviors of various information communication technologies (Ramadhan et al., 2022), this study employs the ECM as the theoretical underpinning to examine how ECM factors (i.e., confirmation, perceived usefulness, perceived enjoyment, and satisfaction) are related to university students’ intention to continue learning online. However, other factors can also affect people’s behavioral intention toward the adoption of information technologies, such as the opinions of prominent individuals (subjective norms; Fishbein and Ajzen, 1975). Since previous studies manifested that the theory of planned behavior (TPB) could also explain people’s continuous intention of learning online (Lee, 2010; Winarno et al., 2020), this study attempts to integrate TPB factors (i.e., subjective norm and PBC) into the EMC model to investigate how these factors can shape student’s continuance intention of online learning at the case of China. We applied structural equation modeling (SEM) to test the proposed research model and hypotheses. Theoretically, the results can fill the conceptual gap between the ECM and the TPB to gain a deeper understanding of students’ continuance intention toward online learning. Practically, the findings can help developers of online learning by identifying the key predictors of users’ continuance intention.

## Literature review

### Expectation-confirmation model

Over the past decades, the ECM has been extensively used to predict user’s satisfaction and continuance behavior in various contexts (Ramadhan et al., 2022). According to the ECM, three factors depend upon a user’s intention to continue using Information Technology (IT), including the level of satisfaction; the extent of the user’s confirmation of expectations; and postadoption expectations, represented by perceived usefulness.

User satisfaction is defined as a positive attitude toward a certain new media technology. Extant research supports that user satisfaction is a critical determinant of people’s continuance intention toward IT. For instance, Kim et al. (2021) found that customer satisfaction positively affects customers’ behavioral continuance intention toward e-services. Chauhan et al. (2021) found that satisfaction positively determined the continuance intentions of the cyber classroom among students and faculties. In this vein, it is expected that students who are satisfied with online learning would have a higher intention to continue use it. Therefore, the following hypothesis was posited:

H1: Students’ satisfaction with online learning positively affects its continuance intention.

Moreover, ECM suggests that user satisfaction is predicted by the other two factors, such as the perceived usefulness of IT and the user’s confirmation of expectations. Confirmation of expectations is defined as a person’s perception of sameness between expectations and actual performance (Bhattacherjee, 2001). According to the ECM, users will be satisfied with an information system if they confirm their expectations (Zuo et al., 2021). Prior studies in online learning also demonstrated that confirmation is positively associated with users’ satisfaction (Lee, 2010; Persada et al., 2021; Ramadhan et al., 2022). As such, this study proposed that:

H2: Students’ confirmation of expectations positively affects their satisfaction with online learning.

Perceived usefulness refers to the degree to which users assume using the technology is useful, which is a chief reason for people to adopt certain technology sometime (Bhattacherjee, 2001). Several prior studies have identified that perceived usefulness and user’s satisfaction are positively correlated (Saxena et al., 2021; Singh and Sharma, 2021). As such, it is predicted that students will be more satisfied with online-learning systems once they perceive the convenience and practicality of the online-learning system (perceived usefulness). Additionally, past studies have revealed that confirmation positively affects users’ perceived usefulness (Alhumaid, 2021). For instance, Wang et al. (2021) found that expectation confirmation occurs through the comparison of expectations with previous experience, which consequently affects people’s perceptions of usefulness. A study by Puriwat and Tripopsakul (2021) also revealed that confirmation was positively associated with the perceived usefulness of e-learning. Thus, the following hypotheses were proposed:

H3: Perceived usefulness positively affects their satisfaction with online learning.

H4: Students’ confirmation of expectations positively affects the perceived usefulness of online learning.

### Linking perceived enjoyment to expectation-confirmation model

Previous studies have identified that perceived enjoyment plays a critical role in expectation confirmation and satisfaction (Winarno et al., 2020). Perceived enjoyment is defined as “the fun and pleasure derived from using Information Technology” (Davis et al., 1992). Mulhem and Almaiah (2021) found that confirmation and perceived enjoyment are positively correlated. That is, people who confirmed their expectations of using an information system were more likely to enjoy using it. Then, the increased enjoyment would lead to a greater intention to adopt the online-learning system (Cavus et al., 2021). Since several studies revealed that perceived enjoyment mediates the relationship between confirmation and satisfaction (Ashrafi et al., 2020), this study posits that:

H5: Perceived enjoyment positively affects students’ satisfaction with online learning.

H6: Students’ confirmation of expectations positively affects their perceived enjoyment of online learning.

### The theory of planned behavior

Additionally, numerous studies have demonstrated that people’ continuance intention toward the adoption of information technologies can be influenced by other behavioral factors, such as the opinions of prominent individuals and other rules (subjective norms; Fishbein and Ajzen, 1975). Thus, this study attempts to integrate TPB factors (i.e., subjective norm and PBC) into the EMC model to investigate how these factors can frame students’ continuance intention of online learning in the context of China. The TPB is a deep-rooted model that predicts human decision-making behavior (Ajzen, 1991). As an extension of the theory of reasoned action (Salamandra, 2012; Du et al., 2020), the TPB recognizes subjective norms and PBC as critical predictors influencing people’s behavioral intention.

Subjective norm, one of the key components of TPB, refers to the perception of important people think what he should or should not perform in particular situation (Fishbein and Ajzen, 1977). According to TPB, a person tends to have higher intentions to engage in certain behaviors if they suppose their social guides like parents and friends reckon such behaviors imperative (Yanovitzky et al., 2006). Extensive previous studies in new media technology have shown that subjective norm positively relates to people’s intention to adopt certain behaviors (Al-Emran et al., 2020; Husain et al., 2021). Based on the aforementioned discussion, it is reasonable to expect that students’ continuance intention toward online learning might be positively influenced by other people’s opinions, particularly those of teachers and respected people. Thus, the following hypothesis was proposed:

H7: Subjective norm positively affects students’ continuance intention toward online learning.

PBC, another key component of TPB, is defined as someone’s perception of easiness or hardness while doing something (Ajzen, 2002). According to TPB, PBC has a positive impact on behavioral intention (Ajzen, 1991). It is widely acknowledged that the perception of control is a key determinant of interactive technology assumptions (Hoffman and Novak, 1996). Prior research in an online-learning context also found that PBC was positively associated with users’ continuance intention (Rahaman et al., 2022). As such, this study hypothesized that:

H8: PBC positively affects students’ continuance intention toward online learning.

**Figure 1:**
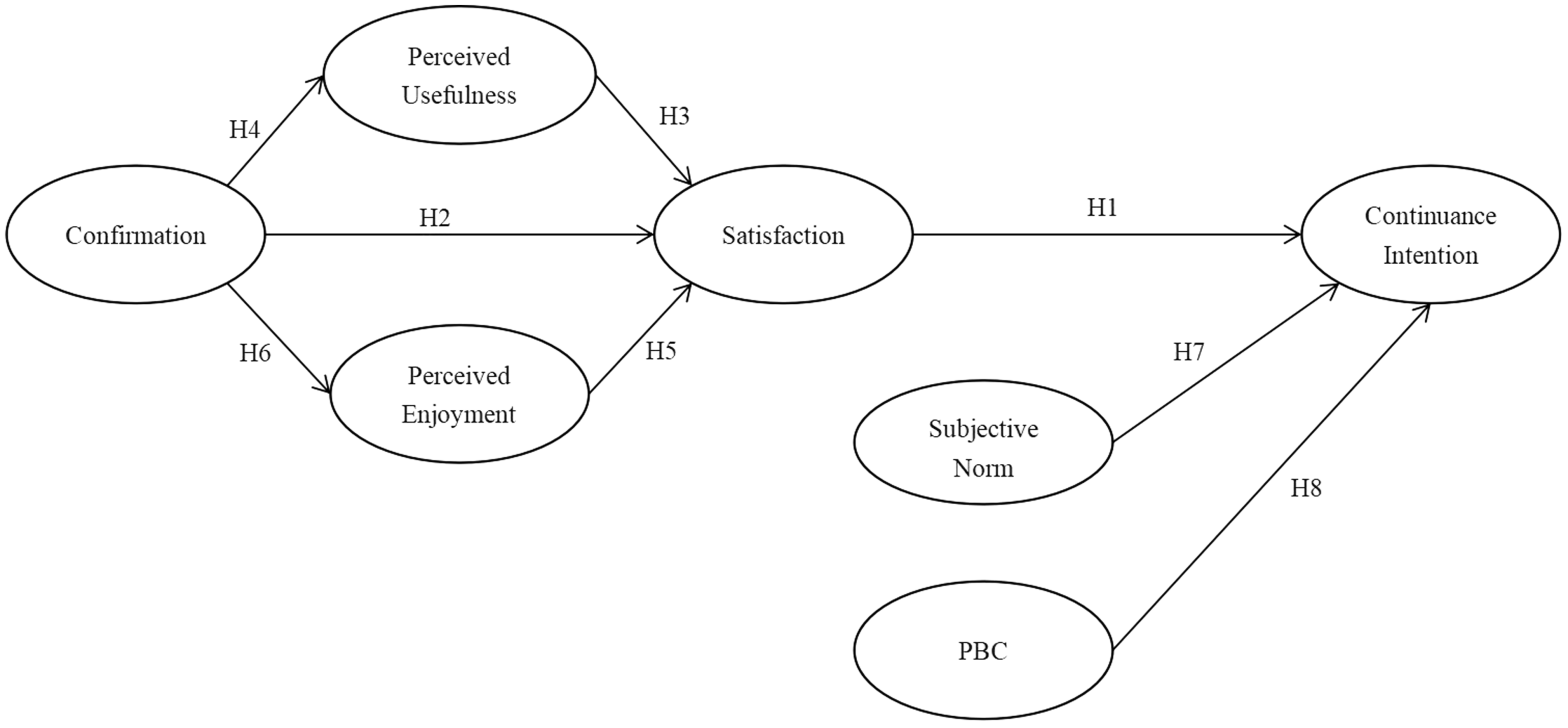
Research model proposed in the study.

The study framework and hypotheses are represented in Figure 1. According to ECM and TPB, the key variables of these two frameworks have been identified, namely confirmation, perceived usefulness, enjoyment, satisfaction, perceived behavioral control, subjective norm, and continuance intention.

## Materials and methods

### Research design

In order to examine the research model and hypotheses, data from a cross-sectional web survey were collected. Data collection was outsourced to one of the largest online survey companies in China.^1^ The survey was administered in November 2020 through convenient sampling. Before starting the survey, the participants were informed of the purpose and contents of the study. All participants provided informed consent that was consistent with the guidelines set by the Declaration of Helsinki (World Medical Association, 2018). To ensure the quality of the sample, we enrolled those participants who full-time university students who have used online-learning platforms. Each respondent completed the survey in around 10 min. In total, 493 Chinese university students responded to the survey. They are mostly female (55.40%) with a mean age of 20.82.

### Measures

The questionnaire contained seven constructs, which were all validated in previous studies. Confirmation was estimated by using a four-item scale adapted from Tang et al. (2014). Participants were asked to indicate how much they are confirmed when using the online-learning method. It has a good reliability of Cronbach’s *α* = 0.771 in the current study. Perceived usefulness and Continuance intention were assessed by three items adapted from Wu and Zhang (2014). Respondents were questioned to indicate their viewpoint about online learning, whether it could improve productivity, performance or not, and their continuance intention toward using online learning. Cronbach’s *α* of perceived usefulness and continuance intention are 0.825 and 0.787, respectively. Perceived enjoyment was measured using two items adapted from Lee (2010). Satisfaction was measured using three items adapted from Chiu et al. (2007). Individuals were required to report their level of satisfaction and perceived enjoyment. Cronbach’s *α* was.725 and 0.791, respectively. Subjective norm and PBC were measured using three items adapted from Lung-Guang (2019). People were asked to report the influence of subjective norm and PBC on online learning. Cronbach’s *α* of subjective norm and PBC are 0.731 and 0.694, respectively. Based on a 5-point Likert scale, respondents indicated their agreement with the above items (1 = strongly disagree, 5 = strongly agree). Table 1 illustrate the detailed measurement items of each included construct.

**Table 1:** Measurement items.

Construct	Code	Measurement items
Confirmation	CF1	My experience with Web-based learning was better than what I expected.
	CF2	The knowledge level provided by Web-based learning was more abundant than what I expected.
	CF3	The learning by using Web system was confirmed with what I expected.
Perceived usefulness	PU1	Using Web-based learning would improve my productivity in learning.
	PU2	Using Web-based learning would improve my academic performance.
	PU3	I find the Web-based learning is useful for me.
Perceived enjoyment	PE1	I use the online-learning tool because it is fun.
	PE2	I use the e-learning tool because I enjoy it.
Satisfaction	SF1	I believe that using Web-based learning is a good idea.
	SF2	I believe that using Web-based learning is advisable.
	SF3	I am satisfied in using Web-based learning.
Subjective norm	SN1	The teachers in my university support the use of Web-based learning.
	SN2	People who are important to me think that I should use Web-based learning.
	SN3	The people whose views I respect support the use of Web-based learning.
Perceived behavioral control	PBC1	I have the knowledge necessary to use Web-based learning
	PBC2	I have control over Web-based learning.
	PBC3	I have the resources necessary to use Web-based learning
Continuance intention	CI1	I intend to continue using the Web-based learning in the future.
	CI2	I will continue using the Web-based learning as much as possible in the future.
	CI3	I will continue using the Web-based learning in the future.

### Data analysis

Followed by the recommendation of Anderson and Gerbing (1988), this study adapted a two-step approach to structural equation modeling. First, for verifying our proposed model, a confirmatory factor analysis was conducted. Then, structural equation model (SEM) was used to investigate the relationships among all constructs. Finally, multiple indices of model fit were examined by Bollen (1989). As part of confirmatory analysis and structural equation modeling, Chi-square, goodness-of-fit index (GFI), adjusted goodness-of-fit index (AGFI), comparative fit index (CFI), and root-mean-square error of approximation (RMSEA) are used to determine the goodness of fit. AMOS software was utilized to analyze the whole process.

## Results

The CFA analysis revealed that the measurement model’s *χ*^2^/df value of 2.539 was below the recommended value of 3.0 (Kline, 2010), which showed a good fit with the data, RMSEA value of 0.056 was smaller than 0.08 (McDonald and Ho, 2002), and the GFI of 0.926, NFI of 0.926 and CFI of 0.953 also surpassed the critical value of 0.9 (Kline, 2010). All are indicated in Table 2. Moreover, the standardized factor loading demonstrated high convergent validity for each of the measurement items, as shown in Table 3. The levels of statistical significance are below 0.05.

**Table 2:** Goodness-of-fit indices and model fits.

	*χ*^2^ (df)	*p*	*χ*^2^/df	CFI	GFI	NFI	RMSEA
Recommended values	N/A	>0.05	<3.0	>0.9	>0.9	>0.9	<0.080
CFA model	378.272 (149)	<0.001	2.539	0.953	0.926	0.926	0.056
Proposed conceptual model	410.696 (159)	<0.000	2.583	0.949	0.912	0.919	0.031

df, degree of freedom; CFI, comparative fit index; GFI, goodness of fit index; NFI, normed fit index; RMSEA, root mean square error of approximation.

**Table 3:** Standardized loadings of indicators in CFA model (*N* = 493).

Construct	CFA model	
	Items	Standardized loadings
Confirmation (CF)	CF1	0.81
	CF2	0.63
	CF3	0.75
	CF4	0.74
Perceived usefulness (PU)	PU1	0.82
	PU2	0.77
	PU3	0.76
Perceived enjoyment (PE)	PE1	0.69
	PE2	0.83
Satisfaction (SF)	SF1	0.81
	SF2	0.64
	SF3	0.80
Subjective norms (SN)	SN1	0.78
	SN2	0.74
	SN3	0.55
Perceived behavioral control (PBC)	PBC1	0.56
	PBC2	0.66
	PBC3	0.76
Continuance intention (CI)	CI1	0.78
	CI2	0.70
	CI3	0.78

By using SEM, this study was able to examine the significance of the integral model; additionally, the significance of the relationships along with variances among the multiple variables was tested too. Our entire model had a high significance level (*χ*^2^ = 410.696; df = 159; *p* < 0.001). Furthermore, the *χ*^2^/df value of 2.583 matched the criteria of less than 3 suggested by Kline (2010). Moreover, based on the statistical analysis, all the values except the adjusted goodness of fit index (AGFI) were within reasonable ranges. Specifically, the goodness of fit index (GFI) of 0.9120 and the normed fit index (NFI) of 0.919, while the comparative fit index (CFI) of 0.949, which were above the acceptable value of 0.9 and 0.95, respectively. Despite the AGFI being under the acceptable level (0.894), it was on the threshold of 0.9 and in previous research, this has been acceptable (Chen et al., 2010). Furthermore, the root mean residual (RMR) and root-mean-squared error of approximation (RMSE) were 0.031 and 0.057, respectively. Since the above values are less than the guidelines of 0.10 for RMR (Steiger, 1990) and 0.08 for RMSEA (McDonald and Ho, 2002), the proposed model was in a good fit with the data. All are indicated in Table 2.

**Figure 2:**
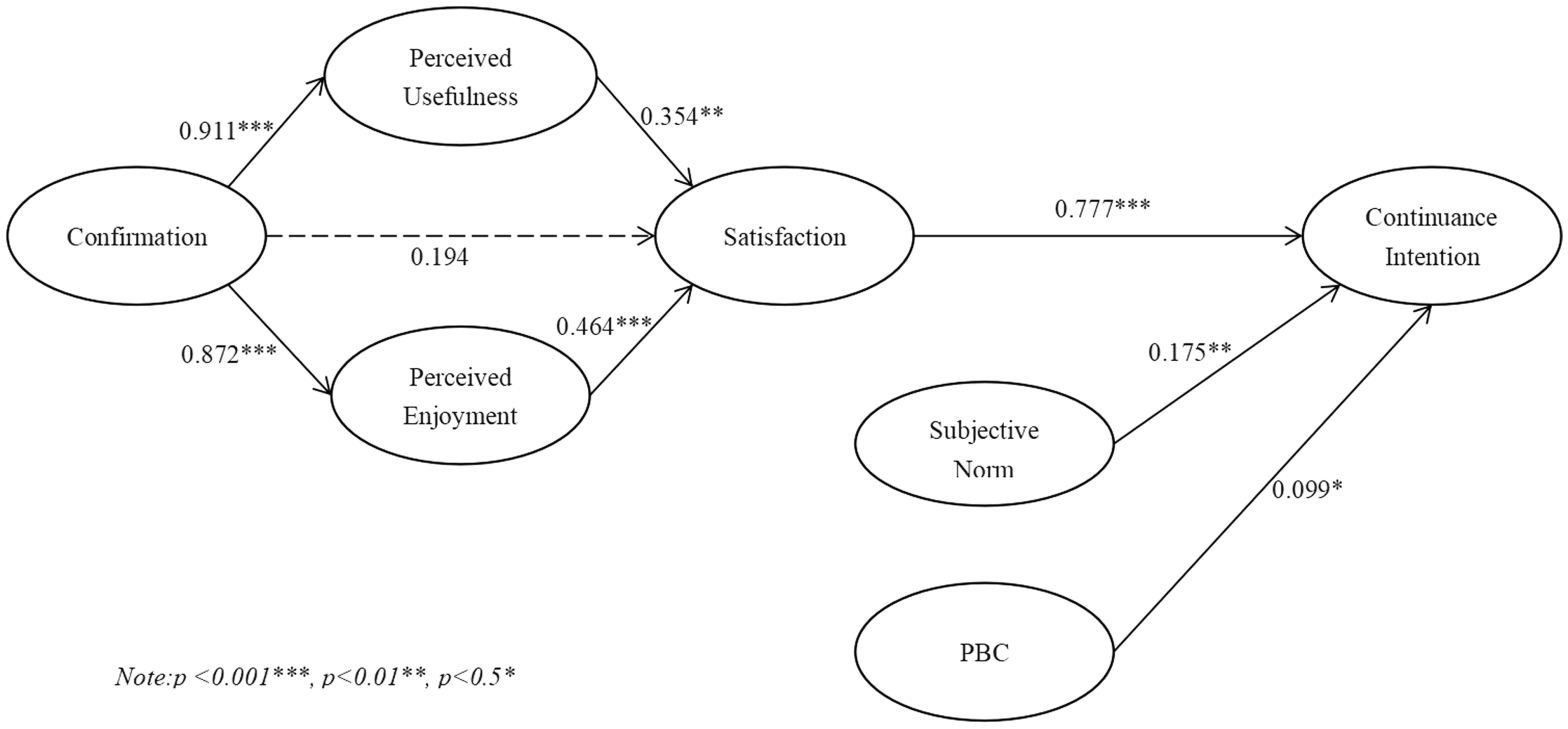
SEM results of the research model.

The path analysis of our hypothesized model is shown in Figure 2. The results show that perceived satisfaction (*β* = 0.777, *p* < 0.001), subjective norms (*β* = 0.175, *p* < 0.001), and PBC (*β* = 0.099, *p* < 0.05) were significantly associated with continuance intention of online learning, indicating that H1, H7, and H8 are supported. Furthermore, the two components of the expectation–confirmation model (ECM), perceived usefulness (*β* = 0.354, *p* < 0.01) and perceived enjoyment (*β* = 0.464, *p* < 0.001), have a significant impact on the satisfaction of online learning, referring to H3 and H5 are testified. Notably, although confirmation of expectations had no direct impact on satisfaction (H2 is not supported), it was positively associated with perceived usefulness (*β* = 0.911, *p* < 0.001) and perceived enjoyment (*β* = 0.872, *p* < 0.001), suggesting that H4 and H6 are supported.

## Discussion

Since the COVID-19 pandemic spread and mutated over the world, online learning becomes an obligatory and emergency choice for college students due to the highly contagious virus which should be taken cautiously (Soria-Barreto et al., 2021). This study focuses on Chinese college students and attempts to determine what factors influence their continuance intention to use online learning during the post-pandemic era.

First of all, we found that confirmation has a significant influence on perceived usefulness, which aligns with the results of previous studies (Cheng, 2019). As reported by the results, a good online-learning experience and abundant knowledge available contribute to a greater level of confirmation, which then improves the students’ perceived usefulness (including productivity and performance) during the learning process. Meanwhile, course information and system quality are the essential impelling causes for continued using online-learning systems (Mustafa and Garcia, 2021). As a result, course quality and service quality should be the top priority to better enhance the infrastructure of online learning. Future studies could focus on the basic needs of students who adopted the online-learning method. Qualitative studies are encouraged to dig out more inner and core factors that influence their continued intention of online learning during the pandemic.

Secondly, it is interesting to find that there was no significant relationship between confirmation and satisfaction. This result is not in line with other studies demonstrating the positive effect of confirmation on satisfaction (Winarno et al., 2020). Nevertheless, the mediation role of perceived enjoyment between confirmation and satisfaction has been supported. Even though students perceived that the way of online learning is much better than they expected, they would not catch themselves with great satisfaction. This may be explained by the distinction between online and offline teaching. Despite the advanced technology of online learning, the vivid interaction and satisfaction derived from face-to-face communication could never be replaced. Thus, it is essential to point out that online learning is not only about the quality of learning content, but also about the interaction between students and teachers which releases a high level of satisfaction. Also, perceived enjoyment is extremely important for using an online-learning system (Muqtadiroh et al., 2019). The happiness gained from online learning could contribute to higher satisfaction. Thus, future researchers may stand out of the existed theories or models and add possible variables to extend original theory. As it in this study, pay more attention to examine how to improve students’ perceived enjoyment and the fun of learning online courses. An experimental method may be able to investigate the differences between well-established platforms (consisting of interactive elements, like compliments with flowers or integration) and general courses (just display the learning contents) on persuading students’ continued online-learning behavior.

Similarly, H3 has been supported, which is consistent with past research guided by ECM (Weng et al., 2015). The result indicates that the level of students’ satisfaction can partially be attributed to the perceived usefulness of online learning. Meanwhile, those with high satisfaction also have a high continuance intention to use online learning, corresponding with the studies of Wang et al. (2021) and Lu and Wang (2019). Undoubtedly, a positive attitude toward online learning would trigger positive emotions. Rajeh et al. (2021) have revealed that students’ satisfaction is the leading force for predicting their continued intention to use online learning. Given that, we should shed light on the appropriate setting of the online curriculum, as well as improve the satisfaction of users to better promote online learning in the future. Future studies could work on the satisfaction variable to find out what influence students’ emotional status and its effects on follow-up behaviors.

Moreover, this study also alleged that subjective norms and PBC have a significant impact on the continuance intention to use online learning. These findings are similar to prior studies which posited that learners’ adaptability and behaviors participating in a practice-oriented focus MOOC rely on these two antecedents (Yang and Su, 2017). More specifically, subjective norm plays a pivotal role in shaping students’ continuance intention. A possible explanation would be that due to the pandemic, people fall into panic, filled with the fear of losing friends and future results in school (Alhumaid et al., 2021). At this moment, the subjective norm has an effect by providing harmonious belief and common faith to help the individual to overcome negative feelings. The support from influential people such as teachers or instructors could largely alleviate the tension of the pandemic. Due to the collective characteristics of China, the results of the present study may differ in other countries. Subsequent research could consider subjective norm as a cultural context, to underestimate its effects on continued online learning among different cultures.

Besides, the pronounced connection between PBC and continuance intention is noted, which is congruent with previous research (Cheon et al., 2012; Gómez-Ramirez et al., 2019). Nonetheless, this impact was smaller than that of other constructs. This result inspires us to endow students with high management and interactive initiative, which would increase the likelihood of students’ continued usage of compulsory online learning (Hadadgar et al., 2016). Also, improving students’ controlled motivation developing throughout the process of online learning could become another way to improve students’ self-efficacy and build up a higher perception of control (Saeed Al-Maroof et al., 2021). Nevertheless, what improves or deteriorates users’ PBC still remains a question in the pandemic online-learning context, and follow-up studies are encouraged to work on this issue.

### Theoretical and practical implications

The present study contributes to research in several ways. Theoretically, this study adds to the existing literature on online learning in the contexts of the COVID-19 pandemic by integrating the ECM and TPB theory. Different from most of previous studies which focused on the ECM constructs and investigated daily online-learning context (Gupta et al., 2020), the current study targeted on the post-pandemic era of COVID-19, and tried to examine the personal and social cognitive variables that influence Chinese college students’ continued usage of online learning in a combined theoretical model. Therefore, our study established a new framework to enrich the exploration of online learning in a public crisis environment. Specifically, we examined the effect of perceived enjoyment between confirmation and satisfaction, which can enhance the existing literature on the ECM. When it comes to the continuous intention, perceived enjoyment should not be neglected, as it reflects ones’ internal thoughts. The interactive characteristics of online-learning interface could increase the perceptions of engagement and enjoyment, and thus their continuance intention (Tsai et al., 2018). In other words, this study applied an emotional lens to understand the integral process of individuals’ intention to consistently use online-learning systems, which aligns with relevant research (Ashrafi et al., 2020). The role of emotion did not limit to positive affects, other feelings such as disappointment or anxiety may also influence the continuance intention in online-learning contexts, which deserve further investigation.

From the practical perspective, recognizing students’ intention to continued use of online learning during the mandatory management has many important implications. Firstly, under the COVID-19 pandemic, online learning is able to boost holistic development owing to school management. Online learning is not only used in the scene of home, but available to school dormitories if students were lockdown there (Soria-Barreto et al., 2021). Online-learning platform has developed theoretical-oriented and practical-oriented courses (Yao et al., 2020), providing multiple choices to better meet the needs of users during the pandemic. Moreover, some scholars highlighted that the quality of online service and course content, and student-teacher interaction are important to predict learners’ continuance intentions in the course of online learning (Li et al., 2021). The findings from this study thus emphasized the importance of interaction design of online learning. If the user feedback is quick enough, the level of students’ confirmation and perceived usefulness will be higher. Furthermore, online-learning platforms could allow different universities to work on the same curriculum design online. Synthesized each school teaching characteristic, online learning lets the students receive the newest and most authoritative study content without geographical boundaries, which would consequently improve their sense of fairness and satisfaction, then exerting positive effect on continued intention to use online learning. Lastly, considering the influence of peers and instructors on students, lecturers could seek for potential methods that increase students’ confidence and then motivate them to continue using online-learning modes, such as setting rewards for those who achieve high scores. It is also important to provide comprehensive training for the students on their master skills in the online-learning system (Winarno et al., 2020). The more proficiency students have in using the system, the higher self-efficacy and controllability they will be.

### Limitations and future research

Our study has several limitations. Firstly, the negative emotions caused by the pandemic are excluded from our study. During the lockdown and quarantine, students may be overwhelmed with a sense of loneliness and stress. These negative emotions could reduce the online-learning intention (Alhumaid et al., 2021). Follow-up studies could examine what and the extent to which these psychological factors caused by COVID-19 influence online learning, both positive and negative emotions, which may improve the knowledge about online learning through an inside and personal perspective. Secondly, our research sample is not completely representative of the general university population, and we also did not take personal characteristics such as demographic variables into consideration, which may cause bias when generalizing the findings. Future research could adopt a longitudinal study and qualitative method to discuss different experiences of online learning, adding age, sex, and different epidemic circumstances into the investigation, which may find valuable results. Thirdly, variables from instructors are not reflected in the proposed model. Apart from students, the teachers’ perspective can be instructive (Sucu and Çakiroğlu, 2022), such as investigating the main factors that directly stimulate their continuous intention to use online-learning method. Therefore, future research could dig in these issues to give a comprehensive understand about online learning and its continuance intention during the pandemic.

## Data availability statement

The raw data supporting the conclusions of this article will be made available by the authors, without undue reservation.

## Ethics statement

Ethical review and approval were not required for the study on human participants in accordance with the local legislation and institutional requirements. The patients/participants provided their written informed consent to participate in this study.

## Author contributions

LL: conceptulization, study design, data collection, and funding. QW: data analysis and first draft. JL: data analysis, review and revise, and coordination. All authors contributed to the article and approved the submitted version.

## Funding

This work was supported by the National Social Science Fund of China for the project (19ZDA332) entitled “Deep Convergence of Media and Innovation Model of Social Governance in the New Era”.

## Conflict of interest

The authors declare that the research was conducted in the absence of any commercial or financial relationships that could be construed as a potential conflict of interest.

## Publisher’s note

All claims expressed in this article are solely those of the authors and do not necessarily represent those of their affiliated organizations, or those of the publisher, the editors and the reviewers. Any product that may be evaluated in this article, or claim that may be made by its manufacturer, is not guaranteed or endorsed by the publisher.

## Footnotes

^1^ https://www.wjx.cn/
